# Circadian Modulation of Protein Catabolism: Insight from a Population Study

**DOI:** 10.3390/nu18101594

**Published:** 2026-05-17

**Authors:** Miriam Lisa Kafader, Sébastien Sare, Martina Zandonà, Rosaria Del Giorno, Maria Luisa Garo, Sandro Bonetti, Luca Gabutti

**Affiliations:** 1 Faculty of Biomedicine, Università della Svizzera Italiana, 6900 Lugano, Switzerland; miriam.lisa.kafader@usi.ch; 2 Service of Family Medicine, Ente Ospedaliero Cantonale, 6962 Viganello, Switzerland; martina.zandona@eoc.ch; 3 Angiology Service, Ente Ospedaliero Cantonale, 6500 Bellinzona, Switzerland; rosaria.delgiorno@eoc.ch; 4 Biostatistic Unit Mathsly Research, 00128 Rome, Italy; marilu.garo@mathsly.it; 5 Istituto di Medicina di Famiglia, Università della Svizzera Italiana, 6900 Lugano, Switzerland

**Keywords:** circadian rhythm, urinary urea excretion, protein metabolism, nocturnal catabolism, blood pressure dipping, ageing, sex difference

## Abstract

Background/Objectives: Urinary urea excretion is a marker of protein catabolism and follows circadian biological rhythms. Although small-scale studies have suggested a diurnal pattern, its population-level characterisation and determinants remain poorly defined. Methods: This cross-sectional study was conducted within the Ticino Epidemiological Stiffness Study (TEST-study), a population-based cross-sectional analysis of 1202 adults (≥18 years) recruited in the Italian-speaking region of Switzerland in 2017–2018. A final analytical sample of 859 participants provided 24 h urine collections divided into diurnal and nocturnal fractions, from which a day-to-night (Day/Night) urea excretion ratio was calculated as the primary outcome. Multivariable linear regression was used to identify independent determinants. Results: Across the cohort, a predominant nocturnal pattern of urinary urea excretion was observed, with a mean Day/Night ratio below 1. In younger women (<40 years), the ratio approached 1, indicating an attenuated Day/Night pattern, whereas older women (>65 years) displayed a significantly more pronounced nocturnal predominance. No comparable age-related trend was observed in men. In multivariable analysis, advancing age, greater nocturnal blood pressure dipping, and higher sodium excretion were independently associated with the Day/Night urea ratio. Conclusions: Urinary urea excretion, a surrogate marker of protein catabolism, exhibits a measurable Day/Night variation, associated with age, sex, and hemodynamic factors. These findings provide insights for chrononutritional strategies aimed at preserving muscle health across lifespan.

## 1. Introduction

The nitrogen balance is a central component of metabolic homeostasis and reflects the equilibrium between protein synthesis and protein breakdown. Throughout the degradation of amino acids, nitrogen is released as ammonia, a neurotoxic compound that cannot safely accumulate in the circulation. To prevent toxicity, ammonia is rapidly converted in the liver into urea via the urea cycle, allowing safe transport and renal excretion of excess nitrogen [1]. Urea constitutes the primary circulating pool of nitrogen, excluding protein-bound nitrogen, and its production parallels the degradation of dietary and endogenous proteins [2]. Most urea is excreted in the urine, while a minor fraction undergoes hydrolysis in the intestinal tract [3]. Consequently, 24 h urinary urea excretion serves as a reliable surrogate marker for protein catabolism and, under steady-state conditions, for dietary protein intake [1].

Protein metabolism is dynamic. In catabolic states, breakdown exceeds synthesis, leading to increased amino acid oxidation and higher urea production, independently of protein intake. Conversely, anabolic states are characterized by net protein accretion and lower ureagenesis [4]. Importantly, such fluctuations occur not only during overt metabolic stress but also under normal physiological conditions. A diurnal variation in urinary urea excretion has been documented in healthy young adults [5,6], and its persistence even under minimal dietary nitrogen intake indicates that intrinsic fluctuations in protein turnover contribute to daily oscillations in urea generation.

Circadian rhythms are endogenous 24 h oscillations that regulate many physiological processes, including hormone secretion [7], metabolism, and renal function [8]. The central pacemaker in the suprachiasmatic nucleus of the hypothalamus synchronizes peripheral clocks in organs such as the kidney [9]. In the kidney, clock genes maintain local rhythmic activity [10], which is further influenced by the light–dark cycle, feeding schedules, and physical activity [11]. These mechanisms generate predictable day–night variations in renal functions, including glomerular filtration rate (GFR), electrolyte excretion, and water handling [12]. Emerging evidence indicates that urea production and urinary urea excretion also follow a circadian pattern. Experimental disruption of clock genes in animal and human cell models alters ureagenesis [10,13], supporting the idea of clock-dependent regulation of nitrogen metabolism.

Urinary urea excretion is further modulated by renal mechanisms. Tubular urea transporters contribute to the urine-concentrating process and are partly regulated by vasopressin. Glucocorticoids and electrolyte disturbances such as hypokalemia can also influence both protein metabolism and urea handling, highlighting the complex interplay between systemic and renal regulation [1].

In addition to circadian rhythms in the kidney, other physiological functions, such as blood pressure, also follow a circadian pattern. Blood pressure typically dips by 10–20% during the night, known as nocturnal dipping [14]. This reduction is associated with better cardiovascular health, while a lack of dipping is linked to an increased risk of target-organ damage, such as left ventricular hypertrophy, microalbuminuria, and cerebrovascular disease [15].

Circadian rhythms and protein metabolism are influenced by demographic factors. Aging is associated with reduced robustness of day–night renal function patterns [16] and vasopressin secretion [7], as well as changes in protein turnover, including anabolic resistance (reduced ability of skeletal muscle to increase muscle protein synthesis in response to anabolic stimuli such as dietary protein, amino acids, or exercise) [17]. A previous study conducted on the same population demonstrated that altered day–night sodium excretion patterns were associated with non-dipping blood pressure behavior in older individuals [18], as well as increased blood pressure and arterial stiffness [19]. Sex further modulates protein metabolism: differences in serum urea concentration between men and women were observed, but could not be explained by variations in body mass index (BMI) or renal function [20], suggesting a potential hormonal contribution. Testosterone promotes muscle protein synthesis [21], whereas estrogens regulate proteolysis [22]. Although young men and women have comparable whole-body protein breakdown when adjusted for lean mass [23], sex-specific differences emerge with aging, particularly after menopause, with elderly women showing relatively higher muscle protein synthesis compared to age-matched men [24].

While small-scale studies support the existence of a circadian rhythm in urea excretion [5,6], population-level characterization is lacking. The present study aims to characterise the day-to-night (Day/Night) distribution of urinary urea excretion in a large population-based cohort and to identify its demographic and hemodynamic determinants.

## 2. Materials and Methods

### 2.1. Study Design

This cross-sectional study is part of the “Ticino Epidemiological Stiffness Study (TEST-study)”, a population-based research project realised in 2017–2018. The TEST-study was conducted in the Italian-speaking part of Switzerland and included 1202 residents aged ≥18 years. Participants were recruited through random sampling from a mailing list provided by the Swiss Federal Statistical Office, with a response rate of 86%.

### 2.2. Ethical Approval

The study was conducted in accordance with the Helsinki Declaration of 1964 and its subsequent revisions, and it was approved by the Swiss Ethics Committee (CE 3115-2016-01718). All participants were asked to provide written informed consent.

### 2.3. Study Sample

In the original study, a total of 1202 participants were recruited and provided with a questionnaire to assess their general health, medical history, dietary habits, physical activity and cardiovascular risk factors. Participants were stratified by age at the time of sample collection into three groups: under 40 years, 40–65 years, and over 65 years. Additionally, the population was stratified by sex into female and male groups.

Each participant provided a single 24 h urine collection divided into two separate fractions: a diurnal and a nocturnal sample, collected in separate containers. The collected volumes were measured, and three urine samples were taken per participant: one from the diurnal fraction, one from the nocturnal fraction, and one from the combined 24 h urine. Collections with a total volume of less than 400 mL or greater than 6000 mL, or with urinary creatinine excretion below 2.5 mmol or above 30.0 mmol in the 24 h urine sample, were considered invalid and excluded from the study. Samples from participants who reported a loss of more than 100 mL of urine were also excluded from the study. Based on the collected urine data, sodium and creatinine concentrations were measured, and excretion of sodium over 24 h was calculated.

Specifically for this study, the urine samples were thawed using a standardised protocol for all samples to measure daytime and nighttime excretion of urea. The samples stored at −80 °C were checked for completeness, thawed for 10 min in a warm water bath, relabeled and analyzed by the EOC Laboratory (Bellinzona and Valli Regional Hospital) for sodium, potassium, urea and creatinine concentrations, using a Hitachi equipped with an indirect ion-selective electrode and a Hitachi 717 (both from Roche Diagnostics, Rotkreuz, Switzerland).

The 24 h urine collection of each participant was divided into two separate fractions: a diurnal and a nocturnal sample, collected in separate containers. The transition between the two fractions was determined by each participant’s individual bedtime. The duration of each collection period was not taken from self-reported sleep times, but was mathematically derived using urinary creatinine excretion as an endogenous time marker. Given that creatinine is generated and excreted at a relatively constant rate throughout the 24 h period—an assumption commonly used in studies converting spot analyte/creatinine ratios to daily excretion [25]—the creatinine concentrations and volumes of each fraction were used to calculate the number of hours corresponding to the diurnal and nocturnal periods for each participant. This approach provides an objective estimate of period duration that is independent of self-reported timing, thereby reducing potential misclassification bias. We subsequently calculated the hourly diurnal and nocturnal excretion rates for urea and the ratio between hourly diurnal and nocturnal excretion rates (Ratio Day/Night).

To evaluate potential association with dietary protein intake, the estimated protein intake per day was calculated from the 24 h urea excretion using the Maroni formula [26], based on the urinary urea nitrogen (UUN). The UUN is defined as the amount of nitrogen in g excreted over 24 h, which was directly calculated from the amount of urea in mmol excreted over 24 h. The Maroni formula further uses body weight and a constant factor to estimate the extra-urinary nitrogen losses.

Pulse wave velocity (PWV) was assessed by both oscillometric (Mobil-O-Graph, IEM GmbH, Stolberg, Germany) and tonometric (Sphygmo-Cor, AtCor Medical, Sydney, Australia) measurements, but only tonometric data were used in this study. Using PWV the vascular age was calculated using a linear regression model derived from a healthy reference sub-sample of the study population (subjects without known cardiovascular risk factors). In this model, chronological age served as the dependent variable, while PWV was the independent variable. Using the resulting regression equation, vascular age was estimated for each participant as the predicted age corresponding to their specific PWV value [27]. As a second variable the difference between estimated age using the regression (vascular age) and the chronological age was determined as Delta Vascular Age. This approach allows for a direct comparison between an individual’s biological arterial stiffness and their actual chronological years.

Using a 24 h ambulatory blood pressure device (Mobil-O-Graph), blood pressure (BP) was measured every 30 min during the day and every hour during the night. From these data, the mean diurnal and nocturnal systolic blood pressure and the standard deviation (SD) of diurnal systolic pressure was calculated. Nocturnal dipping was defined as the percentage of the reduction from diurnal to nocturnal systolic blood pressure. Based on the nocturnal dipping the participants were divided into the groups: riser (<0%), nondippers (0–10%), normal dippers (10–20%), extreme dippers (>20%) [28].

Participants underwent bioelectrical impedance analysis (BIA) (Akern101, Akern Srl, Pontassieve, Florence, Italy) to estimate body composition using electrical currents. Body mass is divided into fat mass (FM) and fat-free mass (FFM), also known as lean body mass. FFM can be further divided into body cell mass (BCM) and extracellular mass (ECM). The parameters of FM, BCM and body mass have all been normalised by the square of height to give the fat mass index (FMI), body cellular mass index (BCMI) and BMI.

**Figure 1:**
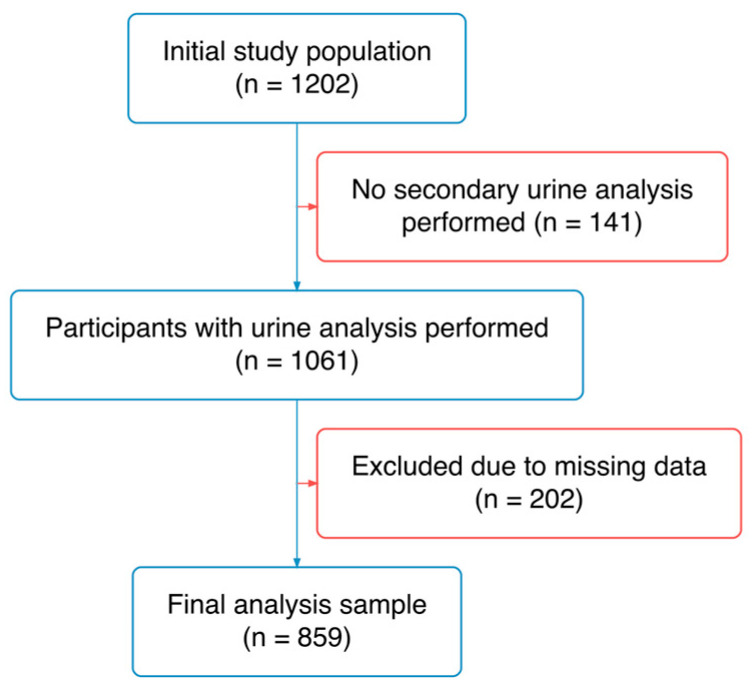
Flowchart showing the participants’ selection procedure.

Of the 1202 participants originally enrolled, urine samples were available only for participants with study numbers 142–1202 (*n* = 1061). From this subset, a further 202 participants were excluded due to missing or invalid data in one or more of the following measures: urine collection criteria (total volume < 400 mL or >6000 mL, urinary creatinine excretion outside the range 2.5–30.0 mmol/24 h, or self-reported urine loss > 100 mL), bioelectrical impedance analysis, ambulatory blood pressure monitoring, or pulse wave velocity. The final analytical sample comprised 859 participants (Figure 1).

### 2.4. Statistical Analysis

Continuous variables are presented as mean and standard deviation (SD), as well as median and interquartile range (25th–75th percentiles), as appropriate. Categorical variables are reported as relative frequencies and percentages. The distribution of continuous variables was assessed using the Shapiro–Wilk test. As all continuous variables significantly deviated from normality, non-parametric methods were used for group comparisons. Specifically, the Mann–Whitney U test was applied for two-group comparisons, while the Kruskal–Wallis test followed by Dunn’s post hoc procedure was used for comparisons involving more than two groups. Interaction effects between age group and sex on the Day/Night ratio of urea were evaluated using factorial linear regression models, with estimation of marginal means and pairwise comparisons. A multivariable linear regression model was subsequently constructed with Day/Night urea ratio as the dependent variable. The following covariates were included: age, sex, BCMI, FMI, BMI, 24 h sodium excretion, 24 h potassium excretion, diurnal systolic blood pressure, nocturnal dipping, Delta Vascular Age, and estimated protein intake. Model assumptions were systematically assessed. 198 Model specification was evaluated using the Ramsey RESET test. Heteroscedasticity was examined using both the Breusch–Pagan and White tests. The normality of residuals was assessed with the Shapiro–Wilk test, and multicollinearity was evaluated using the Variance Inflation Factor. Graphical diagnostics included residual-versus-fitted plots and Q–Q plots. A Bonferroni correction was applied based on the maximum number of categories across all grouping variables used in the analyses. Since the nocturnal blood pressure dipping pattern had the highest number of levels (4 classes), the significance threshold was set at *p* < 0.013 (0.05 ÷ 4) and applied uniformly to all inferential analyses, representing a conservative approach to control the family-wise error rate. All statistical analyses were performed using Stata version 19 (StataCorp LLC, College Station, TX, USA) and R Studio (Version 2025.09.2+418).

## 3. Results

A total of 859 participants were included in the analysis, of whom 484 (56.3%) were female. The mean age of the overall cohort was 50.1 ± 13.5 years. When stratified by sex, significant differences emerged across several clinical and biochemical parameters. Males exhibited a significantly lower Day/Night urea ratio compared with females (0.9 ± 0.2 vs. 1.0 ± 0.2, *p* = 0.029). Conversely, 24 h urinary excretion of sodium, potassium, and urea was markedly higher in males (*p* < 0.001). Males also presented with higher BMI, BCMI, systolic blood pressure, PWV, vascular age, and estimated protein intake (*p* < 0.001). When normalized to body weight, 24 h urea excretion, sodium excretion, and protein intake remained significantly different between males and females (*p* < 0.001, *p* = 0.005, and *p* < 0.001, respectively). Complete results are reported in Table 1.

The Day/Night ratio of urea excretion differed significantly across age categories (*p* = 0.004), showing a progressive decline with advancing age. Older participants displayed a less favorable cardiometabolic and vascular profile. Both BMI and FMI increased significantly across age strata (*p* < 0.001), as did systolic blood pressure. Vascular stiffness demonstrated a pronounced age-related increase, rising from 5.6 m/s in individuals younger than 40 years to 10.1 m/s in those older than 65 years (*p* < 0.001). Vascular age also increased significantly across groups (*p* < 0.001). Significant differences were further observed in 24 h urea, sodium, and potassium excretion, as well as in their bodyweight-adjusted values. Complete results are reported in Table 2.

**Figure 2:**
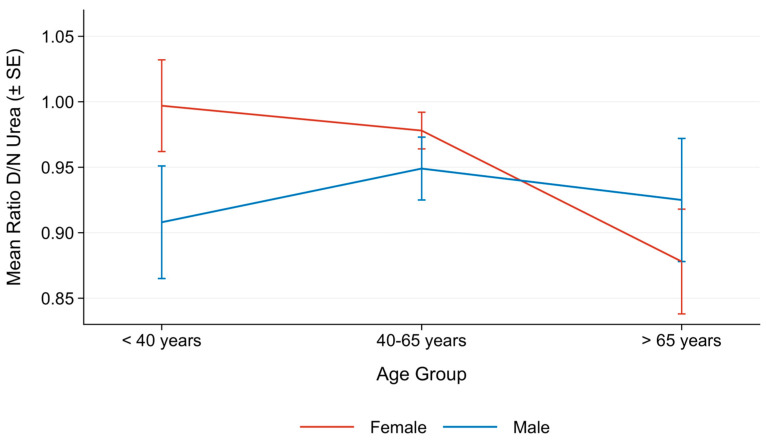
Mean (±SE) Day/Night ratio of urea across age groups and sex. *p*-values refer to comparisons between females and males within each age group. Post hoc comparisons among age groups are also reported if significant.

Analysis of the interaction between age group and sex revealed that a significant sex difference in the Day/Night ratio of urea excretion was present only in the <40 years category (Figure 2). In this subgroup, females exhibited higher values compared to males (0.99 ± 0.02 vs. 0.91 ± 0.02, *p* = 0.001). No significant sex differences were detected in the 40–65 or >65 age groups. Within the female subgroup, the Day/Night urea ratio progressively declined with age, with women older than 65 years showing significantly lower values compared to both younger age groups (<40 and 40–65 years; *p* < 0.001). In contrast, no statistically significant age-related trend was observed among males.

**Figure 3:**
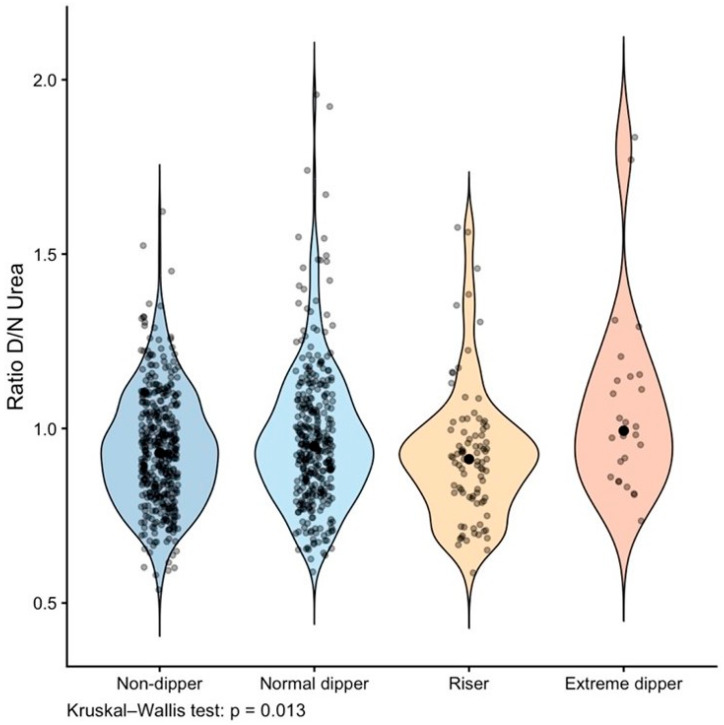
Distribution of the Day/Night ratio of urea according to nocturnal blood pressure dipping pattern. Violin plots display the distribution of the Day/Night urea ratio across dipping pattern. The vertical axis represents the Day/Night ratio of urea. The horizontal width of each violin reflects the estimated data density: wider sections indicate a higher concentration of observations at that value range, whereas narrower sections indicate lower density. Individual data points are overlaid and horizontally jittered to reduce overlap and improve visualization of data concentration.

The Day/Night ratio of urea also differed significantly across nocturnal blood pressure dipping categories (Kruskal–Wallis *p* = 0.013, Figure 3).

Post hoc Dunn’s comparisons revealed significant differences between: normal dippers and risers (*p* = 0.010), extreme dippers and non-dippers (*p* = 0.011), and extreme dippers and risers (*p* = 0.003). Extreme dippers exhibited the highest values (mean 1.1 ± 0.3), whereas non-dippers and risers showed lower values, with normal dippers presenting intermediate values (Table 3).

In the fully adjusted multivariable linear regression model, several variables independently predicted the Day/Night ratio of urea (Table 4). Increasing age was associated with a modest but statistically significant decrease in the outcome (*β* = −0.002, *p* = 0.013). Higher 24 h sodium excretion was independently associated with a greater Day/Night ratio of urea (*β* = 0.0005, *p* < 0.001), as was greater nocturnal BP dipping (*β* = 0.004, *p* < 0.001). In contrast, the estimated protein intake was inversely associated with the outcome (*β* = −0.001, *p* < 0.001). Sex showed a borderline association (*p* = 0.099) and did not retain statistical significance after full adjustment.

## 4. Discussion

### 4.1. Main Findings

In this population-based study, we observed a predominantly nocturnal pattern of urinary urea excretion, with a mean Day/Night ratio below 1, indicating relatively greater hourly urea elimination during the night. This pattern was consistent across male age groups, while in women it became more pronounced with increasing age. The Day/Night urea ratio was independently associated with age and systolic blood pressure dipping. These findings are consistent with a Day/Night distribution of urea excretion at the population level that may reflect relatively greater anabolic activity during the day and catabolic activity at night, although alternative explanations, including circadian variation in renal urea handling, cannot be excluded.

### 4.2. Population Characteristics and External Validity

The demographic, nutritional, body composition, and hemodynamic characteristics of the TEST cohort were consistent with those reported in other Swiss population-based studies, supporting the representativeness of our sample and arguing against relevant selection bias. Twenty-four-hour urinary sodium, potassium, and urea excretion closely matched values described in contemporary Swiss cohorts [29,30,31], and estimated protein intake was comparable to data from the Swiss National Nutrition Survey [32]. Bioimpedance-derived indices, including BMI and FMI, reflected expected values [33] and sex-specific patterns of body composition [34]. Ambulatory blood pressure levels and pulse wave velocity were within reference ranges of central European populations [35,36]. These similarities reinforce the external validity of our findings.

### 4.3. Circadian Pattern of Protein Metabolism

In contrast to earlier small experimental studies reporting higher daytime urea excretion during feeding periods [5,6], our data demonstrate a predominant nocturnal pattern at the population level. Given that 24 h urinary urea is widely accepted as a surrogate marker of whole-body protein catabolism under steady-state conditions [1], this finding is consistent with a relative shift toward net protein degradation at night, although it must be acknowledged that circadian variation in renal urea handling and tubular transport may independently contribute to the observed pattern.

It should also be noted that renal circadian physiology, including clock-gene-driven day/night variation in glomerular filtration rate, tubular urea transport, and vasopressin-mediated urea handling, may independently contribute to the observed nocturnal predominance of urea excretion, independently of systemic protein catabolism [12]. Disentangling renal from metabolic contributions was beyond the scope of the present study and represents an important direction for future research.

While previous investigations were conducted under controlled feeding conditions in small cohorts [5,6], our results derive from a large, free-living population sample, potentially capturing habitual behavioral and physiological patterns. The observed nocturnal predominance of urea excretion may therefore reflect the combined effects of circadian regulation, fasting during sleep, and reduced daytime anabolic stimuli. To better understand these patterns, we explored anabolic and catabolic influences as well as age- and sex-dependent variations.

### 4.4. Anabolic Influences During Daytime

The lower daytime urea excretion observed in our cohort likely reflects the predominance of anabolic stimuli during waking hours. Dietary protein intake, insulin secretion, and physical activity are largely confined to the daytime and represent major drivers of muscle protein synthesis. Postprandial amino acid availability activates mechanistic Target of Rapamycin Complex 1 (mTORC1)-dependent pathways [4], while insulin reduces proteolysis [37] and enhances the anabolic response to feeding [38], particularly under conditions of adequate amino acid supply. The secretion of insulin also follows a circadian pattern [39,40], being higher during the daytime and reduced at night, further favoring anabolic processes during waking hours [39]. Resistance-type muscle activity provides an additional stimulus that can sustain increased protein synthesis for up to 24–48 h after exercise [41]. The temporal clustering of these anabolic signals during the day may therefore promote a more favorable net protein balance, with reduced nitrogen release and consequently lower urea excretion compared with the nocturnal period.

### 4.5. Catabolic Influences During Nighttime

In contrast to the predominance of anabolic stimuli during the daytime, several factors promoting protein breakdown follow circadian patterns that may favor a relatively more catabolic state at night. Glucocorticoids stimulate muscle proteolysis and inhibit protein synthesis [42], with endogenous secretion rising during the late night [43]. Pro-inflammatory cytokines also enhance protein degradation [44,45] and exhibit greater nocturnal activity [46]. In addition, insulin sensitivity declines during the nocturnal phase [47], limiting insulin’s capacity to suppress proteolysis and promote protein synthesis [48], thereby further contributing to net protein breakdown. Together, the circadian alignment of increased glucocorticoid exposure, higher inflammatory activity, and reduced insulin sensitivity during nighttime may promote net protein breakdown, greater nitrogen release, and increased urea production. These mechanisms, combined with the predominance of anabolic stimuli during the daytime, offer a biologically plausible framework for the Day/Night differences in urea excretion observed in our population sample, though direct evidence from our data is lacking, and these mechanisms remain speculative in the absence of direct protein turnover measurements.

### 4.6. Age-Dependent Differences in Day/Night Pattern of Urea Excretion

Age was independently associated with the Day/Night urea ratio, which showed a modest decline with advancing age, indicating relatively greater nocturnal urea excretion or reduced daytime excretion. Post hoc analyses suggested that this pattern was primarily driven by women: participants younger than 40 years differed significantly from those aged 40–64 and ≥65 years, whereas no comparable trend was observed in men.

Several mechanisms may contribute to these findings. Aging is associated with anabolic resistance [17], reduced responsiveness to dietary protein and insulin, and alterations in body composition, including increased fat mass and declining lean mass [49], which may shift protein metabolism toward a more catabolic profile. Circadian rhythms tend to lose amplitude with age [50], potentially modifying Day/Night metabolic regulation and favoring the nocturnal predominance of urea excretion. The apparent sex-specific pattern may further reflect hormonal influences on muscle metabolism and anabolic responsiveness, although this remains speculative and warrants further investigation.

### 4.7. Sex-Dependent Differences in Day/Night Pattern of Urea Excretion

In younger participants under 40 years, significant sex differences in the Day/Night urea ratio were observed. Young women exhibited ratios close to 1, indicating minimal diurnal variation, while young men showed a more pronounced nocturnal predominance of urea excretion. This difference attenuated with age and tended to reverse in participants aged 65 years and older.

The lack of a clear Day/Night pattern in younger women is consistent with a potential modulatory role of female sex hormones on Day/Night protein metabolism. However, menopausal status was not directly assessed in this study, and the age-based inference of hormonal influence remains speculative in the absence of direct endocrine measurements. Estrogens have protective effects on skeletal muscle by attenuating proteolysis [51], modulating inflammatory signaling [52], and supporting repair processes [51]. The decline in estrogen levels after menopause is associated with anabolic resistance [53], reduced muscle mass, and a higher risk of sarcopenia [51,54,55]. A reduced protective effect against nocturnal catabolism may therefore contribute to the lower Day/Night ratio observed in older women. Although causality cannot be inferred and circulating hormones were not measured, the age- and sex-specific patterns suggest a modulatory role of estrogen. Direct hormonal measurements were not available in this study, and the hormonal hypothesis therefore remains speculative and should be tested in future studies with dedicated endocrine assessments.

### 4.8. Association with Blood Pressure Dipping

Blood pressure follows a circadian rhythm, with a physiological nocturnal reduction of approximately 10–20% in systolic values compared with daytime levels, a phenomenon known as nocturnal dipping [14]. In multivariable analysis, the magnitude of systolic BP dipping was independently associated with the Day/Night ratio of urea, although the effect size was modest. Greater nocturnal declines in systolic BP were linked to higher Day/Night ratios, indicating relatively less nocturnal urea predominance, whereas attenuated dipping patterns were associated with lower ratios.

These findings suggest partial coupling between circadian protein metabolism and hemodynamic rhythms. BP dipping reflects coordinated autonomic, hormonal, and renal processes during sleep, and alterations in these systems, characteristic of non-dipping phenotypes, may influence nocturnal protein turnover. Shared upstream factors, including circadian misalignment, variations in nocturnal GFR [56], and altered vasopressin-mediated tubular urea transport, could modulate both BP regulation and urinary urea excretion independently of protein catabolism, and may represent alternative explanations for the observed associations.

### 4.9. Correlation with Nutritional Factors

Although 24 h urinary potassium and total urea excretion were statistically associated with the Day/Night urea ratio, the effect sizes were very small. 24 h potassium excretion showed an inverse association (coefficient: −0.0002, *p* < 0.001), and total urea excretion a positive association (coefficient: 0.00005, *p* < 0.001). These modest correlations suggest that habitual nutritional intake is unlikely to be the primary determinant of the Day/Night distribution of urea excretion. The small effect sizes observed across predictors are consistent with the multifactorial and individually variable nature of protein metabolism, and suggest that no single determinant dominates the Day/Night distribution of urea excretion at the population level. Behavioral and endogenous biological factors may play a larger role than absolute nutrient intake in shaping Day/Night protein turnover.

### 4.10. Clinical Implications, Strengths, and Limitations

This large, population-based study provides novel insights into the Day/Night pattern of urinary urea excretion as a proxy for protein metabolism. The nocturnal predominance of urea excretion, together with age- and sex-specific patterns and associations with BP dipping, highlights a potential interplay between circadian, metabolic, and hemodynamic processes. These findings may inform chrononutritional strategies, timing of protein intake, and understanding of age- or sex-related susceptibility to muscle loss. Strengths include the large, well-characterized cohort, standardized 24 h urine collections, and comprehensive assessment of body composition and ambulatory blood pressure.

The primary limitation of this study is its cross-sectional design, which precludes causal inference and does not allow temporal sequencing between the observed associations to be established; residual confounding cannot be excluded. Further limitations include the use of urinary urea as a surrogate marker of protein catabolism (which is further influenced by renal handling, hydration status, and dietary intake, precluding direct inference about protein turnover), the absence of direct hormone measurements, the lack of standardized meal timing, and the absence of nocturnal GFR data. Although the duration of each collection period was derived mathematically using creatinine excretion rather than self-reported times, this approach relies on the assumption of relatively constant creatinine excretion within each individual over 24 h, an intra-individual assumption that is not affected by inter-individual differences in muscle mass or physical activity. Objective measures of rest periods, such as actigraphy, were not available and would further strengthen the validity of the Day/Night labeling in future studies. Moreover, as the study was conducted in a predominantly Caucasian, Central European cohort with specific dietary habits, lifestyle patterns, and metabolic characteristics, the generalizability of the findings to populations with different ethnic backgrounds, dietary cultures, or environmental contexts may be substantially limited. Behavioural factors such as physical activity, sleep quality, chronotype, and meal timing were not assessed, and it is therefore not possible to determine whether the observed Day/Night variation reflects endogenous circadian regulation, habitual behavioural patterns, or a combination of both. Dietary patterns may further modulate the Day/Night distribution of urinary urea excretion independently of total protein intake. Plant-based diets are associated with lower overall urea production [56]; however, their effect on the Day/Night distribution of urea excretion remains unclear and may depend on meal timing and protein source rather than quantity alone. The present study did not capture detailed dietary patterns or intra-day protein distribution, which represents a limitation and a relevant direction for future chrononutritional research.

## 5. Conclusions

In summary, urinary urea excretion exhibits a measurable Day/Night variation, consistent with a circadian rhythm in protein metabolism at the population level, with relatively greater nocturnal urea excretion. Age and sex modulate this rhythm, with older adults, particularly postmenopausal women, showing a more pronounced nocturnal predominance. Blood pressure dipping was modestly associated with the Day/Night ratio, indicating potential links between metabolic and hemodynamic circadian regulation. These findings highlight the importance of timing in protein metabolism and provide a foundation for future time-based interventions aimed at preserving muscle health across the lifespan.

## Figures and Tables

**Table 1:** Baseline characteristics of the total sample and stratified by sex.

	Total Sample (*n* = 859)	Female (*n* = 484, 56.3%)	Male (*n* = 375, 43.7%)	*p*-Value
Age (years)	50.1(13.5) [50.0; 42.0–59.0]	49.4 (13.7) [50.0; 41.0–58.0]	51.1 (13.2) [51.0; 42.0–60.0]	0.111
Day/Night Ratio Urea	1.0 (0.2) [0.9; 0.8–1.1]	1.0 (0.2) [1.0; 0.8–1.1]	0.9 (0.2) [0.9; 0.8–1.0]	0.029
24 h potassium excretion (mmol)	66.2 (60.6) [61.2; 47.6–77.4]	62.7 (78.3) [56.3; 43.2–70.9]	70.6 (21.6) [68.8; 55.8–83.8]	<0.001
24 h urea excretion (mmol)	400.5 (618.4) [360.3; 279.7–448.8]	364.2 (814.1) [307.8; 247.9–378.1]	447.3 (132.5) [433.2; 353.7–525]	<0.001
24 h sodium excretion (mmol)	149.5 (185.9) [136.5; 96.0–177.7]	137.5 (241.6) [117.0; 86.6–149.5]	164.9 (59.1) [158.4; 124.6–201.9]	<0.001
24 h potassium / kg (mmol/kg)	1.0 (0.9) [0.9; 0.7–1.1]	1.0 (1.2) [0.9; 0.7–1.1]	0.9 (0.3) [0.9; 0.7–1.1]	0.512
24 h sodium / kg (mmol/kg)	2.1 (2.8) [1.9; 1.4–2.5]	2.2 (3.6) [1.8; 1.3–2.5]	2.1 (0.8) [2.0; 1.6–2.5]	0.005
24 h urea / kg (mmol/kg)	5.7 (9.1) [5.2; 4.1–6.2]	5.7 (12.0) [5.0; 3.9–6.1]	5.6 (1.7) [5.5; 4.5–6.6]	<0.001
BCMI (kg/m^2^)	10.4 (2.0) [10.1; 9–11.7]	9.4 (1.5) [9.3; 8.6–10.1]	11.6 (1.9) [11.7; 10.4–12.7]	<0.001
FMI(kg/m^2^)	6.5 (3.3) [5.9; 4.4–7.8]	6.9 (3.5) [6.3; 4.5–8.3]	6.0 (2.8) [5.6; 4.3–7.3]	<0.001
BMI (kg/m^2^)	25.1 (4.4) [24.4; 21.9–27.5]	24.1 (4.5) [23.1; 21.0–26.2]	26.4 (4.0) [26.0; 23.7–28.4]	<0.001
Diurnal systolic pressure (mmHg)	119.1 (11.7) [118.0; 111.0–126.0]	115.8 (11.2) [114.0; 108.0–122.0]	123.4 (11.0) [122.0; 116.0–130.0]	<0.001
SD diurnal systolic pressure (mmHg)	14.0 (4.3) [13.2; 11.0–16.1]	13.5 (4.2) [12.7; 10.7–15.4]	14.5 (4.4) [13.9; 11.4–16.8]	<0.001
Nocturnal dipping (%)	8.5 (6.8) [8.8; 4.2–13]	8.3 (6.7) [8.6; 4.2–12.8]	8.8 (6.9) [9.1; 4.2–13.4]	0.325
PWV (m/s)	7.3 (1.7) [6.9; 6.1–8.3]	7.1 (1.7) [6.7; 5.9–7.9]	7.6 (1.6) [7.4; 6.5–8.5]	<0.001
Estimated Protein Intake (g)	83.9 (108.5) [76.4; 61.3–93.8]	76.2 (142.6) [66.9; 55.5–79]	93.8 (24.0) [91.8; 77.5–108]	<0.001
Protein intake per kg (g/kg)	1.2 (1.6) [1.1; 0.9–1.3]	1.2 (2.1) [1.1; 0.9–1.3]	1.2 (0.3) [1.2; 1–1.4]	<0.001
Delta Vascular Age ^1^ (years)	0.0 (4.5) [0.7; −2.2–3]	0.4 (4.7) [1.2; −2.1–3.7]	−0.6 (4.2) [0.0; −2.6–2.1]	<0.001
Vascular age (years)	50.2 (12.6) [48.2; 40.9–56.8]	49.0 (12.6) [47.4; 39.9–55.4]	51.7 (12.4) [49.6; 42.4–59]	<0.001
Dipper, *n* (%)				
Non-dipper	402 (46.8)	228 (47.1)	174 (46.4)	
Normal dipper	343 (39.9)	190 (39.3)	153 (40.8)	0.967
Riser	88 (10.2)	51 (10.5)	37 (9.9)	
Extreme dipper	26 (3.0)	15 (3.1)	11 (2.9)	

^1^ Delta Vascular Age = Vascular Age—Chronological Age; Results are reported as mean (SD) [median; 25th–75th percentile]. *p*-values refer to comparisons between female and male subjects. Urea excretion and estimated protein intake are reported both as absolute values and normalized.

**Table 2:** Characteristics of the study population stratified by age category.

	<40 Years (*n* = 183, 21.3%)	40–65 Years (*n* = 549, 63.4%)	>65 Years (*n* = 127, 14.8%)	*p*-Value
Day/Night Ratio Urea	1.0 (0.2) [1.0; 0.8–1.1]	1.0 (0.2) [0.9; 0.8–1.1]	0.9 (0.2) [0.9; 0.8–1.0]	0.004
24 h potassium excretion (mmol)	62.7 (23.8) [58.9; 45.1–77.2]	68.2 (71.4) [62.9; 49.8–78.7]	62.4 (44.1) [57.5; 46.8–71.5]	0.017
24 h urea excretion (mmol)	384.8 (140.4) [362.3; 275.6–471.3]	422.2 (765.3) [371.1; 289.5–456.8]	329.1 (145.4) [305.7; 246.3–369.6]	<0.001
24 h sodium excretion (mmol)	147.6 (62.4) [140.8; 99.9–182.6]	153.8 (227) [136.8; 96.2–179]	133.4 (72.5) [122.9; 91.7–161.7]	0.037
24 h potassium / kg (mmol/kg)	0.9 (0.3) [0.9; 0.7–1.1]	1.0 (1.1) [0.9; 0.7–1.1]	0.9 (0.7) [0.8; 0.6–1.0]	0.031
24 h sodium / kg (mmol/kg)	2.1 (0.9) [2.1; 1.6–2.7]	2.2 (3.4) [1.9; 1.4–2.4]	1.9 (1.1) [1.7; 1.3–2.2]	0.003
24 h urea / kg (mmol/kg)	5.5 (1.7) [5.2; 4.3–6.4]	6.0 (11.2) [5.3; 4.3–6.3]	4.7 (2.3) [4.3; 3.5–5.6]	<0.001
BCMI (kg/m^2^)	10.4 (1.9) [10.0; 9–11.7]	10.5 (2.0) [10.2; 9–11.8]	10.0 (2.0) [9.7; 8.6–10.8]	0.014
FMI (kg/m^2^)	5.9 (2.8) [5.3; 4–7.1]	6.6 (3.3) [6.0; 4.5–7.7]	7.2 (3.7) [6.2; 4.7–8.9]	0.003
BMI (kg/m^2^)	24.0 (4.1) [23.5; 21.1–25.6]	25.2 (4.4) [24.5; 22.1–27.6]	26.2 (4.8) [25.8; 22.6–28.9]	<0.001
Diurnal systolic pressure (mmHg)	116.1 (10.0) [114.0; 110.0–121.0]	118.5 (11.7) [117.0; 110.0–126.0]	125.8 (11.9) [124.0; 117.0–134.0]	<0.001
SD diurnal systolic pressure (mmHg)	12.8 (3.4) [12.5; 10.7–14.5]	13.8 (4.1) [13.2; 10.8–15.8]	16.4 (5.1) [15.9; 12.9–19.1]	<0.001
Nocturnal dipping (%)	8.4 (6.6) [8.7; 4.0–12.4]	8.8 (6.7) [9.2; 4.5–13.3]	7.2 (7.5) [7.8; 3.0–13.0]	0.128
PWV (m/s)	5.6 (0.7) [5.5; 5.1–6.1]	7.2 (1.0) [7.0; 6.5–7.9]	10.1 (1.3) [9.9; 9.1–11.1]	<0.001
Estimated Protein Intake (g)	81.0 (26.3) [76.0; 61.1–96.9]	87.8 (134.1) [78.9; 63.3–94.7]	71.3 (26.1) [67.8; 56.2–79]	<0.001
Protein intake per kg (g/kg)	1.2 (0.3) [1.1; 1.0–1.3]	1.2 (2.0) [1.1; 0.9–1.3]	1.0 (0.4) [0.9; 0.8–1.2]	<0.001
Delta Vascular Age ^1^ (years)	−4.7 (4.5) [−4.3; −7.2–1.3]	1.8 (3.2) [2.0; −0.1–3.9]	−1.0 (4.1) [−0.9; −3.6–1.8]	<0.001
Vascular age (years)	36.2 (3.3) [35.9; 34.4–38]	49.6 (6.8) [48.9; 44.6–53.2]	72.7 (8.0) [71.3; 66.2–77.8]	<0.001
Dipper, *n* (%)				
Non-dipper	91 (49.7)	252 (45.9)	59 (46.5)	
Normal dipper	68 (37.2)	229 (41.7)	46 (36.2)	0.523
Riser	18 (9.8)	51 (9.3)	19 (15.0)	
Extreme dipper	6 (3.3)	17 (3.1)	3 (2.4)	

^1^ Delta Vascular Age = Vascular Age—Chronological Age; Results are reported as mean (SD) [median; 25th–75th percentile], except different indications.

**Table 3:** Day/Night ratio of urea by dipping pattern.

	Non-Dipper	Normal Dipper	Riser	Extreme Dipper
Day/Night Ratio Urea	0.9 (0.2) [0.9; 0.8–1.0]	1.0 (0.2) [0.9; 0.8–1.1]	0.9 (0.2) [0.9; 0.8–1.0]	1.1 (0.3) [1.0; 0.9–1.1]

Results are reported as mean (SD) [median; 25th–75th percentile].

**Table 4:** Multivariable linear regression analysis with Day/Night ratio of urea as the dependent variable.

Variables	Coefficient (SE)	*p*-Value	95% CI
Age (years)	−0.002 (0.001)	0.013	(−0.003; −0.0003)
Sex (base: F)	−0.027 (0.016)	0.099	(−0.058; 0.005)
BCMI (kg/m^2^)	−0.009 (0.007)	0.191	(−0.023; 0.005)
FMI (kg/m^2^)	−0.008 (0.006)	0.166	(−0.020; 0.003)
BMI (kg/m^2^)	0.004 (0.005)	0.387	(−0.005; 0.015)
24 h sodium excretion (mmol)	0.0005 (0.0001)	<0.001	(0.0002; 0.0007)
24 h potassium excretion (mmol)	0.0004 (0.0003)	0.211	(−0.0002; 0.0010)
Diurnal systolic pressure (mmHg)	0.0004 (0.0008)	0.673	(−0.001; 0.002)
Nocturnal dipping	0.004 (0.0009)	<0.001	(0.002; 0.006)
Delta Vascular Age	0.001 (0.002)	0.537	(−0.003; 0.005)
Estimated Protein Intake (g)	−0.001 (0.0002)	<0.001	(−0.001; −0.001)

SE: Standard Error, CI: Confidence Interval; Body composition variables (BCMI, FMI, BMI) are included as covariates in the model, providing implicit adjustment for body size.

## Data Availability

Study data are obtainable from the authors upon reasonable request.

## Abbreviations

The following abbreviations are used in this manuscript:

TEST-study	Ticino Epidemiological Stiffness Study
Day/Night	Day-to-night
BMI	Body mass index
EOC	Ente Ospedaliero Cantonale
UUN	Urinary urea nitrogen
PWV	Pulse wave velocity
BP	Blood pressure
SD	Standard deviation
BIA	Bioelectrical impedance analysis
FM	Fat mass
FFM	Fat-free mass
BCM	Body cell mass
ECM	Extracellular mass
FMI	Fat mass index
BCMI	Body cellular mass index
SE	Standard error
CI	Confidence interval
mTORC1	Mechanistic Target of Rapamycin Complex 1

## References

[1] Weiner I.D., Mitch W.E., Sands J.M. (2015). Urea and Ammonia Metabolism and the Control of Renal Nitrogen Excretion. Clin. J. Am. Soc. Nephrol..

[2] Lima C., Macedo E. (2018). Urinary Biochemistry in the Diagnosis of Acute Kidney Injury. Dis. Markers.

[3] Visek W.J. (1978). Diet and cell growth modulation by ammonia. Am. J. Clin. Nutr..

[4] Bröer S., Bröer A. (2017). Amino acid homeostasis and signalling in mammalian cells and organisms. Biochem. J..

[5] Steffee W.P., Anderson C.F., Young V.R. (1981). An Evaluation of the Diurnal Rhythm of Urea Excretion in Healthy Young Adults. J. Parenter. Enter. Nutr..

[6] el-Khoury A.E., Ajami A.M., Fukagawa N.K., Chapman T.E., Young V.R. (1996). Diurnal pattern of the interrelationships among leucine oxidation, urea production, and hydrolysis in humans. Am. J. Physiol.-Endocrinol. Metab..

[7] Hofman M.A., Swaab D.F. (1994). Alterations in circadian rhythmicity of the vasopressin-producing neurons of the human suprachias-matic nucleus (SCN) with aging. Brain Res..

[8] Firsov D., Bonny O. (2010). Circadian regulation of renal function. Kidney Int..

[9] Allada R., Bass J. (2021). Circadian Mechanisms in Medicine. N. Engl. J. Med..

[10] Lin R., Mo Y., Zha H., Qu Z., Xie P., Zhu Z.J., Xu Y., Xiong Y., Guan K.L. (2017). CLOCK Acetylates ASS1 to Drive Circadian Rhythm of Ureagenesis. Mol. Cell.

[11] Solocinski K., Gumz M.L. (2015). The Circadian Clock in the Regulation of Renal Rhythms. J. Biol. Rhythm..

[12] Firsov D., Bonny O. (2018). Circadian rhythms and the kidney. Nat. Rev. Nephrol..

[13] Gumz M.L. (2016). Molecular basis of circadian rhythmicity in renal physiology and pathophysiology. Exp. Physiol..

[14] Pickering T.G. (1990). The clinical significance of diurnal blood pressure variations. Dippers and nondippers. Circulation.

[15] Sachdeva A., Weder A.B. (2006). Nocturnal Sodium Excretion, Blood Pressure Dipping, and Sodium Sensitivity. Hypertension.

[16] Mohandas R., Douma L.G., Scindia Y., Gumz M.L. (2022). Circadian rhythms and renal pathophysiology. J. Clin. Investig..

[17] Wall B.T., Gorissen S.H., Pennings B., Koopman R., Groen B.B.L., Verdijk L.B., van Loon L.J.C. (2015). Aging Is Accompanied by a Blunted Muscle Protein Synthetic Response to Protein Ingestion. PLoS ONE.

[18] Giorno R.D., Troiani C., Gabutti S., Stefanelli K., Puggelli S., Gabutti L. (2020). Impaired Daytime Urinary Sodium Excretion Impacts Nighttime Blood Pressure and Nocturnal Dipping at Older Ages in the General Population. Nutrients.

[19] Giorno R.D., Ceresa C., Gabutti S., Troiani C., Gabutti L. (2020). Arterial Stiffness and Central Hemodynamics are Associated with Low Diurnal Urinary Sodium Excretion. Diabetes Metab. Syndr. Obes. Targets Ther..

[20] Liu Q., Wang Y., Chen Z., Guo X., Lv Y. (2021). Age- and sex-specific reference intervals for blood urea nitrogen in Chinese general population. Sci. Rep..

[21] Bhasin S., Wang C., Chandra M.S., Gagliano-Jucá T., Jasuja R. (2026). Mechanisms of Testosterone’s Anabolic Effects on Muscle and Function: Controversies and New Insights. Endocr. Rev..

[22] Lu L., Tian L. (2023). Postmenopausal osteoporosis coexisting with sarcopenia: The role and mechanisms of estrogen. J. Endocrinol..

[23] Fujita S., Rasmussen B.B., Bell J.A., Cadenas J.G., Volpi E. (2007). Basal muscle intracellular amino acid kinetics in women and men. Am. J. Physiol.-Endocrinol. Metab..

[24] Smith G.I., Mittendorfer B. (2016). Sexual dimorphism in skeletal muscle protein turnover. J. Appl. Physiol..

[25] Johner S.A., Boeing H., Thamm M., Remer T. (2015). Urinary 24-h creatinine excretion in adults and its use as a simple tool for the estimation of daily urinary analyte excretion from analyte/creatinine ratios in populations. Eur. J. Clin. Nutr..

[26] Masud T., Manatunga A., Cotsonis G., Mitch W.E. (2002). The precision of estimating protein intake of patients with chronic renal failure. Kidney Int..

[27] Pasini M., Zandonà M., Garo M.L., Bozzini C., Cinieri F., Giorno R.D., Gabutti L. (2025). Air Pollution, Body Composition, and Vascular Age in Southern Switzerland: A Cross-Sectional Population Study. J. Clin. Med..

[28] Filippone E.J., Foy A.J., Naccarelli G.V. (2023). Controversies in Hypertension III: Dipping, Nocturnal Hypertension, and the Morning Surge. Am. J. Med..

[29] Chelbi S.T., Gianini J., Gagliano V., Theiler K., Alonzo G.L., Marot P., Ackermann D., Durrer I., Beuschlein F., Suter P. (2024). Swiss Salt Study 2, second survey on salt consumption in Switzerland: Main results. Food Risk Assess. Eur..

[30] Petrovic D., Bankir L., Ponte B., Pruijm M., Corre T., Ghobril J.P., Bouatou Y., Ackermann D., Vogt B., Bochud M. (2023). The urine-to-plasma urea concentration ratio is associated with eGFR and eGFR decline over time in a population cohort. Nephrol. Dial. Transplant..

[31] Bochud M., Jenny-Burri J., Pruijm M., Ponte B., Guessous I., Ehret G., Petrovic D., Dudler V., Haldimann M., Escher G. (2018). Urinary Cadmium Excretion Is Associated with Increased Synthesis of Cortico- and Sex Steroids in a Population Study. J. Clin. Endocrinol. Metab..

[32] Inanir D., Kaelin I., Pestoni G., Faeh D., Mueller N., Rohrmann S., Sych J. (2021). Daily and meal-based assessment of dairy and corresponding protein intake in Switzerland: Results from the National Nutrition Survey menuCH. Eur. J. Nutr..

[33] Petrovic D., Younes S.E., Pruijm M., Ponte B., Ackermann D., Ehret G., Ansermot N., Mohaupt M., Paccaud F., Vogt B. (2016). Relation of 24-hour urinary caffeine and caffeine metabolite excretions with self-reported consumption of coffee and other caffeinated beverages in the general population. Nutr. Metab..

[34] Kyle U.G., Kossovsky M.P., Genton L., Pichard C. (2007). Overweight and obesity in a Swiss city: 10-year trends. Public Health Nutr..

[35] Azizzadeh M., Karimi A., Breyer-Kohansal R., Hartl S., Breyer M.K., Gross C., Boutouyrie P., Bruno R.M., Hametner B., Wassertheurer S. (2024). Reference equations for pulse wave velocity, augmentation index, amplitude of forward and backward wave in a European general adult population. Sci. Rep..

[36] Pusterla L., Radovanovic D., Muggli F., Erne P., Schoenenberger A.W., Schoenenberger-Berzins R., Parati G., Suter P., Lava S.A.G., Gallino A. (2022). Impact of Cardiovascular Risk Factors on Arterial Stiffness in a Countryside Area of Switzerland: Insights from the Swiss Longitudinal Cohort Study. Cardiol. Ther..

[37] Rooyackers O.E., Nair K.S. (1997). Hormonal Regulation of Human Muscle Protein Metabolism. Annu. Rev. Nutr..

[38] Garlick P.J., Grant I. (1988). Amino acid infusion increases the sensitivity of muscle protein synthesis in vivo to insulin. Effect of branched-chain amino acids. Biochem. J..

[39] Stenvers D.J., Scheer F.A.J.L., Schrauwen P., la Fleur S.E., Kalsbeek A. (2019). Circadian clocks and insulin resistance. Nat. Rev. Endocrinol..

[40] Perelis M., Marcheva B., Ramsey K.M., Schipma M.J., Hutchison A.L., Taguchi A., Peek C.B., Hong H., Huang W., Omura C. (2015). Pancreatic β cell enhancers regulate rhythmic transcription of genes controlling insulin secretion. Science.

[41] Wolfe R.R. (2006). Skeletal Muscle Protein Metabolism and Resistance Exercise. J. Nutr..

[42] Mitch W.E., Goldberg A.L. (1996). Mechanisms of Muscle Wasting—The Role of the Ubiquitin–Proteasome Pathway. N. Engl. J. Med..

[43] Dickmeis T. (2009). Glucocorticoids and the circadian clock. J. Endocrinol..

[44] Hasselgren P.O., Fischer J.E. (1999). Counter-regulatory hormones and mechanisms in amino acid metabolism with special reference to the catabolic response in skeletal muscle. Curr. Opin. Clin. Nutr. Metab. Care.

[45] Sharma B., Dabur R. (2020). Role of Pro-inflammatory Cytokines in Regulation of Skeletal Muscle Metabolism: A Systematic Review. Curr. Med. Chem..

[46] Shimba A., Ikuta K. (2020). Glucocorticoids Regulate Circadian Rhythm of Innate and Adaptive Immunity. Front. Immunol..

[47] Gibson T., Jarrett R. (1972). Diurnal Variation in Insulin Sensitivity. Lancet.

[48] Rasmussen B.B., Fujita S., Wolfe R.R., Mittendorfer B., Roy M., Rowe V.L., Volpi E. (2006). Insulin resistance of muscle protein metabolism in aging. FASEB J..

[49] Palmer A.K., Jensen M.D. (2022). Metabolic changes in aging humans: Current evidence and therapeutic strategies. J. Clin. Investig..

[50] Kessler K., Pivovarova-Ramich O. (2019). Meal Timing, Aging, and Metabolic Health. Int. J. Mol. Sci..

[51] Oxfeldt M., Dalgaard L.B., Farup J., Hansen M. (2022). Sex Hormones and Satellite Cell Regulation in Women. Transl. Sports Med..

[52] Pellegrino A., Tiidus P.M., Vandenboom R. (2022). Mechanisms of Estrogen Influence on Skeletal Muscle: Mass, Regeneration, and Mitochondrial Function. Sports Med..

[53] Rennie M.J. (2009). Anabolic resistance: The effects of aging, sexual dimorphism, and immobilization on human muscle protein turnoverThis paper is one of a selection of papers published in this Special Issue, entitled 14th International Biochemistry of Exercise Conference—Muscles as Molecular and Metabolic Machines, and has undergone the Journal’s usual peer review process. Appl. Physiol. Nutr. Metab..

[54] Menzies C., Bowtell R., Shur N., Brook M. (2026). Menopause, Female Sex Hormones, Skeletal Muscle Mass and Muscle Protein Turnover in Humans. J. Cachexia Sarcopenia Muscle.

[55] Geraci A., Calvani R., Ferri E., Marzetti E., Arosio B., Cesari M. (2021). Sarcopenia and Menopause: The Role of Estradiol. Front. Endocrinol..

[56] Xu K., Cui X., Wang B., Tang Q., Cai J., Shen X. (2020). Healthy adult vegetarians have better renal function than matched omnivores: A cross-sectional study in China. BMC Nephrol..

